# Sir A. Halliday

**Published:** 1840-01

**Authors:** 


### SIR ANDREW HALLIDAY, M.D., F.R.S. E.

Since our last publication, the profession has to regret the loss of a very estimable member, in the person of Sir A. Halliday, deputy-inspector-general of army hospitals. He died at his seat near Dumfries. Sir A. Halliday was the author of several works well known to the profession, the most recent of which we reviewed in a recent number. We hope to be soon able to give a fuller notice of Sir A. Halliday's active and useful life.

